# Challenges in exome analysis by LifeScope and its alternative computational pipelines

**DOI:** 10.1186/s13104-015-1385-4

**Published:** 2015-09-07

**Authors:** Erinija Pranckevičiene, Tautvydas Rančelis, Aidas Pranculis, Vaidutis Kučinskas

**Affiliations:** Department of Human and Medical Genetics, Faculty of Medicine, Vilnius University, Santariskiu str. 2, LT-08661 Vilnius, Lithuania

**Keywords:** LifeScope, Exome analysis pipeline, GATK, Mapping of color-space sequencing data, MAQ, SHRiMP, BFAST, Annovar, Interpretation of genomic variants

## Abstract

**Background:**

Every next generation sequencing (NGS) platform relies on proprietary and open source computational tools to analyze sequencing data. NGS tools for Illumina platforms are well documented which is not the case with AB SOLiD systems. We applied several computational and variant calling pipelines to analyse targeted exome sequencing data obtained using AB SOLiD 5500 system. Our investigated tools comprised proprietary LifeScope’s pipeline in combination with open source color-space competent mapping programs and a variant caller. We present instrumental details of the pipelines that were used and quantitative comparative analysis of variant lists generated by LifeScope’s pipeline versus open source tools.

**Results:**

Sufficient coverage of targeted regions was achieved by all investigated pipelines. High variability was observed in identities of variants across the mapping programs. We observed less than 50 % concordance of variant lists produced by approaches based on different mapping algorithms. We summarized different approaches with regards to coverage (DP) and quality (QUAL) properties of the variants provided by GATK and found that LifeScope’s computational pipeline is superior. Fusion of information on mapping profiles (pileup) at genomic positions of variants in several different alignments proved to be a useful strategy to assess questionable singleton variants.

**Conclusions:**

We quantitatively supported a conclusion that Lifescope’s pipeline is superior for processing sequencing data obtained by AB SOLiD 5500 system. Nevertheless the use of alternative pipelines is encouraged because aggregation of information from other mapping and variant calling approaches helps to resolve questionable calls and increases the confidence of the call. It was noted that a coverage threshold for variant to be considered for further analysis has to be chosen in data-driven way to prevent a loss of important information.

## Background

High throughput next-generation sequencing (NGS) has become widely used in practical life-science areas for whole genome and exome sequencing or targeted studies aimed at the identification of deleterious disease-causing genomic variants or in general population genetics studies [1, 2]. Accurate interpretation of sequencing results depends on proper laboratory work and a computational pipeline used in the analysis of NGS data. Vendors of each platform provide proprietary computational software tools to perform analysis of sequencing data obtained from their equipment. In addition, there are many open source programs designed by the research community [3, 4]. Software tools designed for the Illumina platform are the most documented. Alternatively, software tools for the Life Technologies SOLiD platform are not widely discussed in the scientific literature although SOLiD still appears to be the sequencing platform of choice in many research centers [5, 6].

Illumina platform is based on sequencing by synthesis and is using letter-based nucleotide encoding. SOLiD platform is employing a different, ligation based, sequencing strategy and uses color-space encoding. In the SOLiD approach each observed DNA base (A, C, G, or T) is encoded by a color label defining an order in which two consecutive nucleotides appear in a read [7]. Two-base encoding greatly facilitates identification of sequencing errors because each base is interrogated twice by ligation chemistry. This strategy increases confidence that observed variations at specific genomic locations are true single nucleotide variants.

**Fig. 1:**
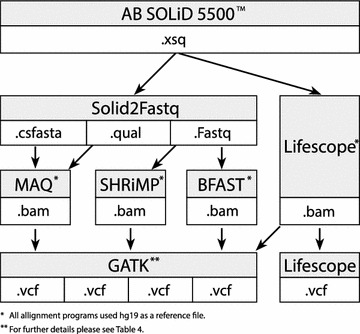
Schema comprising an investigated workflow of exome analysis by LifeScope and the alternative pipeline. This schema represents exome’s computational pipeline steps that were applied in the study. The LifeScope’s pipeline includes proprietary programs to perform alignment and variant calling. Alternatively we applied another four approaches based on combinations of LifeScope, SHRiMP, MAQ, BFAST mapping programs and GATK modules for variant calling, using the same exomes as with Lifescope program

To align color-space reads to a letter-based reference, the letter-space aligners cannot be used without appropriately transforming either the reads or the reference [8, 9]. The color-space competent alignment tools often utilized in practice are Blat-like Fast Accurate Search Tool (BFAST), Bowtie, Short Read Mapping Package (SHRiMP) and Mapping and Assembly with Qualities (MAQ) [10, 11]. However, for mapping and variant calling in SOLiD sequencing data the proprietary LifeScope software by Life Technologies is usually used [5, 12, 13]. Only a very small fraction of reports in literature discussed the LifeScope’s pipeline with respect to its alternatives [5, 12]. Our study attempts to fill this gap by performing a comparative analysis of popular color-space competent open source tools and the proprietary LifeScope program within a framework of LITGEN project (Genetic diversity of the population of Lithuania and changes of its genetic structure related with evolution and common diseases) [14]. The major contributions of our study consists of:

1. *Comparative analysis of the effects of mapping programs on the outcome of variant calling* We analyzed color-space competent mapping programs LifeScope, MAQ, SHRiMP and BFAST using near default settings. The mapping programs produce aligned BAM files that are input to a variant calling procedure by GATK. The same variant calling algorithm was applied to all BAM files and produced lists of variants that were different from each other. We aimed to determine the best variant calling approach out of investigated LifeScope, LifeScope-GATK, MAQ-GATK, BFAST-GATK and SHRiMP-GATK combinations. Schema of our experimental setup is presented in Fig. 1. Quality (QUAL) and coverage depth (DP) of the variant reported by GATK were used as criteria to compare the approaches.
2. *Evidence in favor of using an aggregated list of Single Nucleotide Polymorphysms (SNPs) in exomes identified by different approaches in search of possible causative variant-phenotype relationship* Annovar was used for variant annotation [15, 16]. Our analysis of annotated variants led to the conclusion that pooling the variants identified by LifeScope and alternative pipelines is more informative in search of possibly damaging variants than using LifeScope’s pipeline alone.
3. *Detailed description of computational pipeline for analysis of color-space coded targeted exome sequencing data* The pipeline comprises all steps from the mapping of raw reads up to the calling of the genomic variants in the exome. We utilize Genome Analysis Toolkit (GATK) for variant calling [17, 18]. GATK tasks and their parameters that worked with LifeScope’s BAM files and the alternative to LifeScope mapping programs are described.

Exomes were sequenced at the department of Human and Medical Genetics, Vilnius University with the Life Technologies SOLiD 5500 system using the TargetSeqTM Exome Enrichment Kit without Exact Call Chemistry (ECC). The sequenced fragment read lengths were of 75 bp in color-space coding. A summary statistics of exome sizes of 48 sequenced population samples is as follows: mean = 44.79 million reads and quartiles were Q25 = 27.75, Q50 = 40.50 and Q75 = 58.50 million reads. To demonstrate our analysis strategy we use family exomes of proband, father and mother having 63, 31 and 28 million sequenced reads respectively. The targeted regions of the exome comprise 195,282 regions in total and consists of 37,268,825 bases. A workstation and a computing cluster (4 nodes, 48 cores) running CentOS 5.6 operating system were used to analyze exomes.

## Results

### Mapping

**Table 1:** Percentages of the exome target regions by the mapping programs in family exomes at the cutoffs of 5×, 10×, 15× and 20×

Coverage	5$\times$ (%)	10$\times$ (%)	15$\times$ (%)	20$\times$ (%)
LifeScope	94.28	91.29	87.69	83.56
SHRiMP	93.97	89.92	84.94	79.07
MAQ	90.91	85.17	78.82	71.92
BFAST	86.72	78.76	70.47	62.04

It is recommended by GATK creators that 80 % of targeted regions are covered at least by 20$\times$ in order to achieve good results by GATK. In clinical setting it is recommended to have coverage of at least 30$\times$ [19]. The percentages of the target regions in all trio samples covered by 5$\times$, 10$\times$, 15$\times$ and 20$\times$ by each mapping method are summarized in Table 1. The best coverage of the target exome regions was achieved by LifeScope and the lowest was achieved by BFAST (78 % by 10$\times$) comparing favorably with the coverage in the published exome analysis study of intellectual disability which had 75 % of targeted regions covered at least by 10$\times$ [20].

**Fig. 2:**
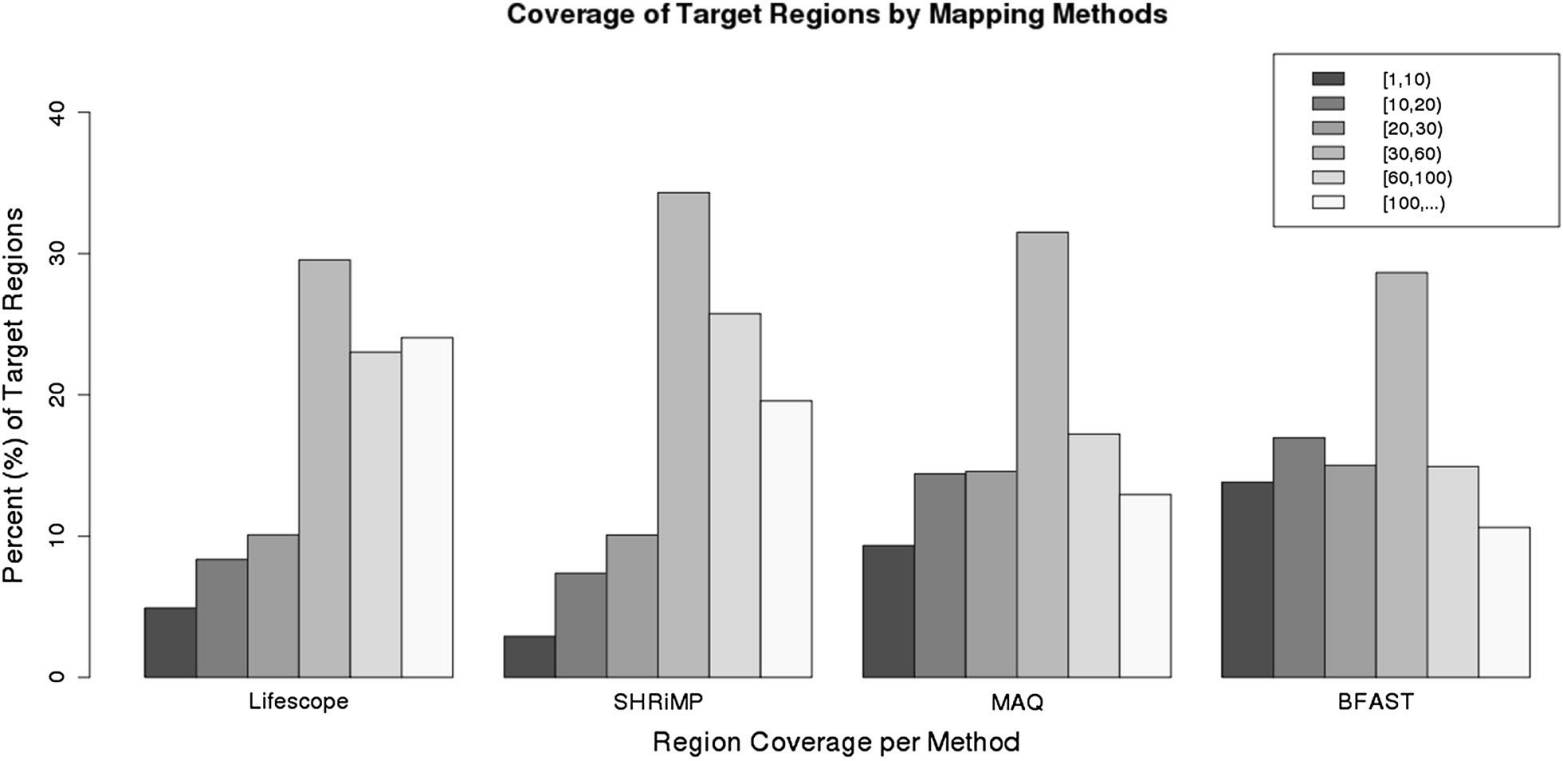
Coverage of target regions by mapping methods. Average coverage of the targeted exome regions by the mapped reads in family exomes is shown. Five coverage intervals that we created to assess mappings of different methods are presented in the legend. They comprise intervals of [1,10), [10,20), [20,30), [30,60), [60,100) and equal and higher than 100. Each individual barplot shows a percentages of the targeted regions falling into a coverage category for each mapping method: LifeScope, SHRiMP, MAQ and BFAST. The targeted regions are mostly covered by 30–60 reads in all mapping methods

All mapping methods covered 97 % of the targeted regions. How the methods comapre to each other in mapping is presented in Fig. 2. The largest fraction of the targeted exome regions are covered by 30–60 reads. LifeScope and SHRiMP produced better coverage than MAQ and BFAST. We computed which fraction of regions of the low-coverage (less than 20 reads) by LifeScope are covered better by other mapping programs. SHRiMP improved coverage on 6 % and MAQ on 1 % of those regions. Analysis of the agreement between the individual aligners shows that alternative aligners can map only negligible fraction of the reads unmapped by LifeScope. If compared to each other, then MAQ can map about 28 % of the reads unmapped by BFAST and about 19 % of the reads unmapped by SHRiMP. BFAST can align 12 % and SHRiMP can align 19 % of the reads unmapped by MAQ.

### Characterizing different approaches by transition/transversion (Ti/Tv) ratio

**Table 2:** Transition transversion ratio in different variant calling approaches

Approach	LifeScope	LifeScope-GATK	MAQ-GATK	SHRiMP-GATK	BFAST-GATK
Ti/Tv	2.62	2.45	2.59	2.3	2.68

To verify if we obtained reliable variant calls we used transition/transversion ratio. This ratio represents a standard to which empirical data should be compared to ensure that the called variants are not random and agree with the natural fundamental variability in human genome. Validated human SNP data showed that a rate between frequency of transitions (T$>$C, A$>$G) in genomes to frequency of transversions (T$>$A, T$>$G, C$>$A, C$>$G) in human genome is 2.1 and in it’s coding part (exome) this rate is higher—around 2.8 [17, 21]. If Ti/Tv ratio is lower such as 0.5–1, then it might mean that variants are found at random or there are sequencing artifacts. In our study Ti/Tv ratio of variant lists produced by the different approaches indicated that identified variants were not random. Ti/Tv ratio in the analyzed exome data by different methods was close to 2.2–2.7 as shown in Table 2. The agreement between the methods with respect to Ti/Tv ratio was high.

### Variant calling results

**Table 3:** Counts of SNPs identified in the family exomes present in the datasets of known variants

	LifeScope	LifeScope-GATK	MAQ-GATK	SHRiMP-GATK	BFAST-GATK
Proband					
Total	*38,626*	*60,313*	*49,483*	* 67,669*	*43,536*
dbsnp138	37,312	58,342	48,538	65,185	42,909
1000G	35,511	54,745	46,706	62,778	42,030
esp6500	31,319	29,745	26,041	27,386	25,169
In target regions	* 21,837*	*19,530*	*16,958*	*17,082*	*15,856*
dbsnp138	20,892	18,869	16,707	16,768	15,688
1000G	19,504	17,954	16,260	16,304	15,321
esp6500	17,894	16,469	14,912	14,891	14,138
Mother					
Total	*35,267*	*43,831*	* 28,032*	* 38,711*	* 26,898*
dbsnp138	34,075	42,778	27,641	38,337	26,626
1000G	32,455	40,316	26,472	36,856	26,170
esp6500	29,095	26,101	17,909	23,348	19,139
In target regions	* 20,995*	* 17,697*	* 12,566*	* 15,477*	*12,722*
dbsnp138	20,099	17,296	12,470	15,359	12,623
1000G	18,806	16,459	12,143	14,939	12,384
esp6500	17,281	15,099	11,163	13,658	11,469
Father					
Total	*36,350*	* 46,822*	* 36,798*	* 42,486*	* 27,949*
dbsnp138	35,106	45,723	36,209	42,193	27,669
1000G	33,381	42,939	34,777	40,782	27,204
esp6500	29,765	26,728	22,177	24,458	19,715
In target regions	* 21,254*	* 17,936*	* 14,972*	* 15,760*	* 13,033*
dbsnp138	20,348	17,531	14,827	15,661	12,931
1000G	19,015	16,668	14,447	15,296	12,692
esp6500	17,457	15,323	13,326	14,042	11,756

For each exome and the approach a total number of all identified SNPs and the SNPs that are only in the targeted regions are shown in italic

**Table 4:** Agreement between variant calling approaches on all called SNPs

Exome	Proband	Mother	Father
Total number of SNP variants *union* of all approaches	86,840	55,738	68,915
Number of SNP variants identified by all approaches *common*	*21,687*	*13,995*	*11,483*
Fraction of common variants in total identified by LifeScope (%)	56	40	32
LifeScope-GATK (%)	32	44	25
MAQ-GATK (%)	44	50	31
SHRiMP-GATK (%)	32	36	27
BFAST-GATK (%)	50	52	41
Number of SNP variants and fraction of total *specific* to LifeScope	2936 (7.6 %)	4543 (12.9 %)	4246 (11.7 %)
LifeScope-GATK	5251 (8.7 %)	4468 (10.2 %)	4555 (9.7 %)
MAQ-GATK	1943 (3.9 %)	1074 (3.8 %)	1369 (3.7 %)
SHRiMP-GATK	14,276 (21.1 %)	2210 (5.7 %)	1644 (3.9 %)
BFAST-GATK	1097 (2.5 %)	574 (2.1 %)	9851 (35.2 %)

By common are denoted variants that have been identified by all approaches. By specific are identified variants that were identified exclusively by one approach. The percentages are computed as fraction of the total number of all variants shown in Table 3 identified by that method

Regardless of a targeted nature of exome sequencing experiment important variants can be found outside the boundaries of the targeted regions [22]. Therefore we used all identified variants in our study. It is expected that majority of the identified variants have already been found and documented in public databases. We assessed how many variants found in the analyzed exomes are present in dbSNP, 1000Genomes and ESP6500 databases. These counts are shown in Table 3. Most of the identified variants (96–98 %) were included in dbSNP138, thus validating the used approaches. Largest proportions of variants were found by LifeScope-GATK and SHRiMP-GATK. The least number of variants was found by BFAST-GATK. A proportion of variants found by each method correlates with the number of original reads available for mapping and also the mapping efficiency of the alignment program.

We observed a moderate concordance between the variant lists. The concordance was measured by counting overlapping genomic positions of variants between the methods computed by *intersect* tool in BEDtools [23]. Table 4 shows numbers and fractions of variants found by all approaches and singleton variants specific to methods. All methods agree on 30–50 % of identified variants. This is consistent with the results of the study in which a concordance in variant calling approaches was investigated for Illumina platform and it was found to be less than 60 % [24]. LifeScope and LifeScope-GATK consistently identified around 10 % of variants unique only to those methods. In MAQ-GATK approach this fraction was around 4 %. Fractions of method-specific variants in SHRiMP-GATK and BFAST-GATK are not consistent across the exomes. Variability in variant identities across the methods arises due to differences in BAM files caused by differences in mapping algorithms and mapping qualities.

**Fig. 3:**
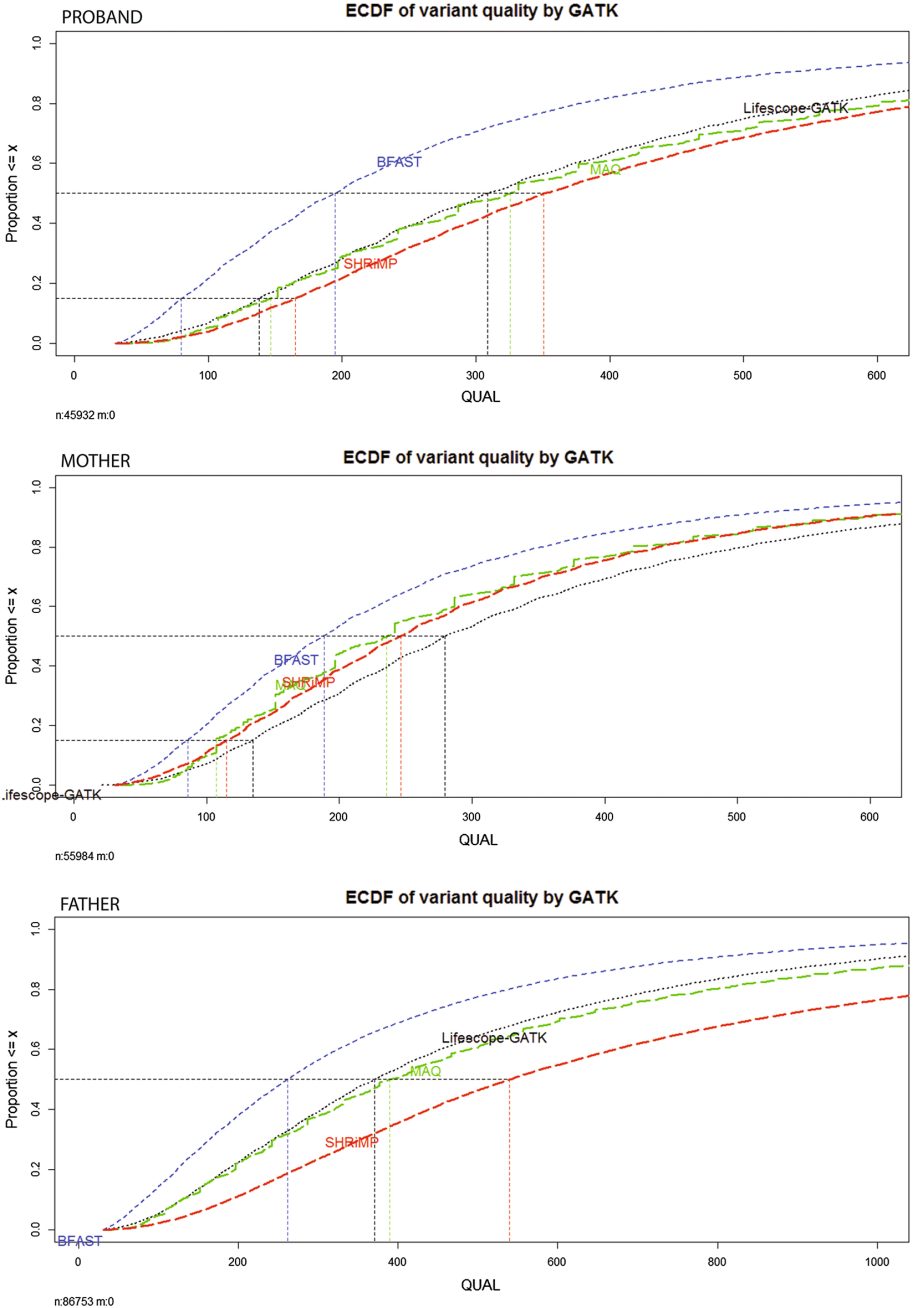
Empirical cumulative distribution function (ECDF) of variant quality (QUAL) property assigned by GATK for variants identified in alignments produced by different mapping programs. To compute ECFD only variants that have been identified by all approaches together were used. ECDF’s of different alignments are color-coded: BFAST by *blue line*, LifeScope-GATK by *black*, MAQ by *green* and SHRiMP by *red*. Panels correspond to the family exomes. ECDF plots of QUAL per method in proband exome are on the *top*, the mother exome is in the *middle* and exome of the father is on the *bottom*. Median QUAL value of LifeScope-GATK consistently apears around 300 in all exomes. For BFAST-GATK it is around 200. Other approaches differ across the exomes

**Table 5:** Summary of coverage [depth of coverage (DP) assigned to a variant by GATK] for variants called by the different approaches in family exomes

Proband variants	All variants per method			Common 21,687 variants		Variants in COSMIC	
Identification method	Coverage quartiles Q25,Med,Q75	Total #	Sites % ≥ 8×	Coverage quartiles Q25,Med,Q75	Sites % ≥ 8×	Coverage quartiles Q25,Med,Q75	Total #
LifeScope	12,25,46	38,626	87	16,28,46	92	11,28,51	89
LifeScope-GATK	7,14,24	60,313	75	14,21,30	95	16,22,35	21
MAQ-GATK	7,13,23	49,483	74	10,17,25	86	25,33,64	15
SHRiMP-GATK	6,16,34	67,669	72	21,32,50	98	15,52,120	22
BFAST-GATK	7,13,22	43,536	73	11,18,27	89	7,8,10	10

Mother variants	All variants per method			Common 13,995 variants		Variants in COSMIC	
Identification method	Coverage quartiles Q25,Med,Q75	Total #	Sites % ≥ 8×	Coverage quartiles Q25,Med,Q75	Sites % ≥ 8×	Coverage quartiles Q25,Med,Q75	Total #
LifeScope	8,15,26	35,267	76	12,20,30	89	7,16,28	101
LifeScope-GATK	6,10,16	43,831	65	10,15,21	90	10,23,27	13
MAQ-GATK	6,10,15	28,032	61	6,10,15	66	25,32,61	6
SHRiMP-GATK	6,11,17	38,711	68	10,14,20	88	17,20,34	13
BFAST-GATK	5,9,14	26,898	58	2,12,16	74	5,6,8.5	6

Father variants	All variants per method			Common 11,483 variants		Variants in COSMIC	
Identification method	Coverage quartiles Q25,Med,Q75	Total #	Sites % ≥ 8×	Coverage quartiles Q25,Med,Q75	Sites % ≥ 8×	Coverage quartiles Q25,Med,Q75	Total #
LifeScope	8,16,29	36,350	78	12,21,31	89	8,15,31.5	99
LifeScope-GATK	6,11,18	46,822	68	11,16,22	90	14,23,30	12
MAQ-GATK	6,10,17	36,798	66	8,12,18	76	34,57,94	5
SHRiMP-GATK	7,12,20	42,486	74	12,18,25	94	17,21,21	4
BFAST-GATK	6,9,15	27,949	61	7,11,17	73	8,12,17	93

Coverage is summarized as median and 1*st* and 3*rd* quartile showing a central tendency. This coverage tendency is presented for: all called variants, common variants called by all methods and approach-specific variants in COSMIC. An additional quality measure—a fraction of variants—covered at least by 8 reads is shown for each method for all and common variants

Depth of coverage (DP) and variant quality (QUAL) properties are assigned by GATK to the called variants. DP represents number of reads that overlap in the genomic position of a variant. QUAL is Phred encoded score assigned to the variant by GATK showing call quality and it can be very large. We assume that a better variant calling approach produces variants with high DP and QUAL values. To compare the DP and QUAL across the methods we used the variants simultaneously identified by all used methods. Figure 3 illustrates per-method per exome differences in QUAL property by means of its empirical distribution functions. The best QUAL values were achieved by SHRiMP-GATK, followed by MAQ-GATK, followed by LifeScope-GATK and the last was BFAST-GATK. The overall result for the DP property is presented in Table 5. With regards to DP a highest variant coverage is achieved by LifeScope. Variants produced by both SHRiMP-GATK and LifeScope-GATK have higher median coverage than MAQ-GATK and BFAST-GATK.

With respect to both DP and QUAL properties of the variant, SHRiMP-GATK ranks first and LifeScope-GATK second. Variant calls by MAQ-GATK do not have high coverage (DP value) nevertheless they have high quality. In proband exome 46.54 % of the calls had higher or equal DP in BFAST-GATK versus 53.46 % of the calls in LifeScope-GATK, in the mother exome it was 25.02 versus 74.98 % and in the father exome it was 27.32 versus 72.68 %. Overall results that we observed suggest that LifeScope mapping program is superior in mapping color-space data.

### Variant annotation

Variant annotations have been performed using Annovar [15]. It is the most widely used software tool for interpretation of genomic variants found in high-throughput sequencing data. Annotation was performed using *table_annovar.pl* script, which generates an Excel compatible file with integrated information for a given list of variants. For variant interpretation it is important that the used pipeline identifies as many deleterious variants as possible.

**Fig. 4:**
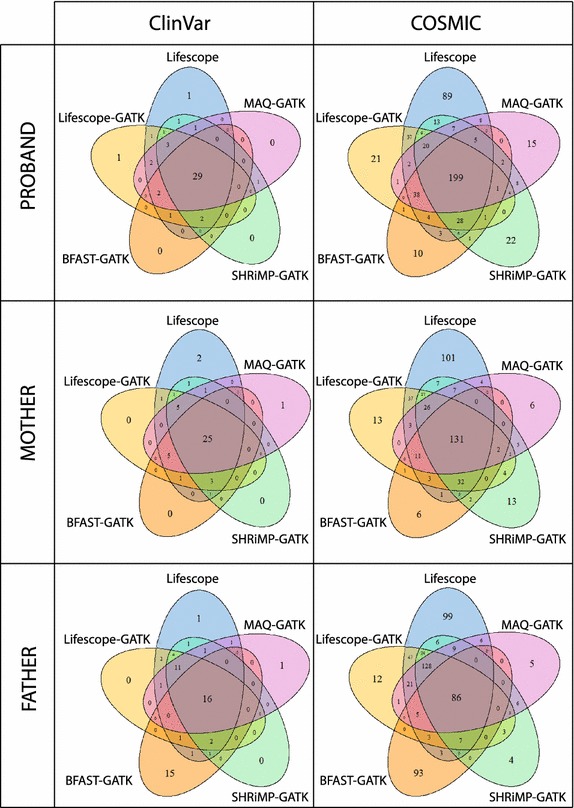
Agreement between different variant calling approaches with respect to called SNPs that are present in ClinVar and COSMIC databases. Venn diagrams show how much different approaches agree in identifying harmful variants. The middle area of each diagram shows number of variants common to all methods. The Venn diagram *leafs* show number of variants specific to each method. On the left are diagrams representing SNPs in ClinVar database and on the right is distribution of identified SNPs present in COSMIC database. The ittop part of the figure shows diagrams of proband, the itmiddle represents mother and the *bottom* represents father. Substantial agreement between the methods was observed on pathogenic and drug response ClinVar variants

ClinVar is a public archive that provides reports of relationships among medically important variants and phenotypes. Data to ClinVar streams from OMIM, GeneReviews, dbSNP and also from direct submissions by scientists. Database represents 19,774 genes which include 149,202 variants from 248 submitters [25]. COSMIC (Catalog of Somatic Mutations in Cancer) database is designed to store and display somatic mutation information and contains information relating to human cancers - publications, samples and mutations. COSMIC database describes over 2500 cancer disease classifications, from 47 primary tissue types and represents full literature curation of 136 genes and 12,542 cancer genomes [26]. We analyzed how many harmful variants from ClinVar and COSMIC were identified by each investigated variant calling approach and how these approaches complemented each other in detecting important deleterious variants. Summary of this analysis is presented in Table 6. Figure 4 illustrates an agreement of the variant calling approaches in detecting those deleterious variants. Largest number of deleterious variants is detected by LifeScope’s pipeline. Pipelines based on MAQ and BFAST are similar to each other in terms of their performance.

**Table 6:** SNPs annotated in ClinVar and COSMIC databases per method and per person

	LifeScope	LifeScope-GATK	MAQ-GATK	BFAST-GATK
Proband				
ClinVar total	1328	1261	1116	1150
Deleterious variants	44;34pat + 10drug	42;33pat + 9drug	38;29pat + 9drug	34;27pat + 7drug
Deleterious at 15×	32;23pat + 9drug	38;28pat + 10drug	24;19pat + 5drug	21;15pat + 6drug
Loss going to 15×	27 %	9.5 %	37 %	38 %
COSMIC total	468	366	310	299
COSMIC variants at 15×	349	238	195	168
Loss going to 15×	25 %	35 %	37 %	44 %
Mother				
ClinVar total	1288	1170	841	947
Deleterious variants	47;38pat + 9drug	41;33pat + 8drug	38;30pat + 8drug	36;30pat + 6drug
Deleterious at 15×	20;15pat + 5drug	30;24pat + 6drug	11;10pat + 1drug	11; 8pat + 3drug
Loss going to 15×	57 %	27 %	71 %	69 %
COSMIC total	395	292	197	194
COSMIC at 15×	231	124	73	63
Loss going to 15×	42 %	52 %	63 %	68 %
Father				
ClinVar total	1248	1126	950	959
Deleterious variants	43;36pat + 7drug	37;31pat + 6drug	31;26pat + 5drug	36;30pat + 6drug
Deleterious at 15×	19;16pat + 3drug	26;22pat + 4drug	12;11pat + 1drug	11;9pat + 2drug
Loss going to 15×	56 %	30 %	61 %	69 %
COSMIC total	444	340	270	197
COSMIC at 15×	288	176	122	74
Loss going to 15×	35 %	37 %	55 %	62 %

Total number of identified variants in ClinVar is shown together with the number of patogenic (pat) and drug response (drug) variants. Counts of high confidence variants covered at least by 15$\times$ are presented for both: ClinVar and COSMIC. A fraction of total variants which would be lost by going to a higher coverage is presented by a percentage

A variant of high confidence has to have high coverage. Table 6 presents calculation of information loss if only taking into consideration the deleterious variants covered by 15$\times$. About $\sim$10–15$\times$ is assumed to be a minimum coverage for a confident call [20]. Our results show that in cases of exomes having smaller coverage high-threshold coverage filters may lead to information losses up to 50 %. Lesser loss was noted in LifeScope’s pipeline. The biggest loss of detected variants by constraining the coverage was in BFAST-GATK and MAQ-GATK approaches.

Overlap of the methods in terms of their detected deleterious variants is illustrated in Fig. 4. Almost every pathogenic and drug response variant was identified by either one or several out of four alternative approaches and LifeScope. Few singleton pathogenic variants were specific to LifeScope and LifeScope-GATK pipelines. Just a single variant was detected solely by MAQ-GATK. Comparison of approaches with respect to variants in COSMIC showed that there are cases in which alternative pipelines are calling an important variant, which did not gain enough coverage or quality during the processing by other pipelines. Use of several alternative variant calling pipelines helps to resolve questionable variant calls.

### Differences between the used variant calling approaches

We compared performance of proprietary versus open source based pipeline in exome analysis using a near default setting and observed only moderate agreement of different methods on SNP calls. Similar agreement of variant calling pipelines on data produced by Illumina platform was already noted [24]. It can be argued that the agreement of different methods with respect to SNP calls can be improved by parameter tuning in mapping and variant calling phases.

Ideally for the purposes of comparison, the compatible parameters of mapping programs would be set to equal values to generate BAM files for variant calling. Such equalization is very difficult in practice and may require a reverse-engineering of the mapping programs. LifeScope operates using dozens of parameters. A majority of these parameters do not have obvious counterparts in the open source aligners. Other variation which is difficult to control arises from indexing. In BFAST and SHRiMP indexing of the reference by default spaced seeds will be different from the indexing scheme used by LifeScope leading to differing alignment of reads in BAM files affecting the variant calling. Variant calling features affected by mapping can be explored through the GATK variant annotations.

Using variants identified by HaplotypeCaller (see step 8 in Table 7) GATK builds a confident variant call prototype based on a multivariate Gaussian mixture modelling. Model parameters are estimated using variant annotations computed from data in BAM file of called variants present in the dbSNP database of known variants. Final variants are called by applying GATK VariantRecalibrator task (see step 9 in Table 7). Variant annotations—quality (qual), depth (DP), Fisher Strand (FS), root mean square of Mapping Quality of reads supporting a variant call (MQ), quality by depth (QD), Mann–Whitney–Wilcoxon Rank Sum tests MQRankSum, ReadPosRankSum, BaseQRankSum and ClippingRankSum—characterize low level properties of variants from information in BAM file. By comparing values of these annotations the major differences between variants identified by different approaches ( Lifescope-GATK, MAQ-GATK, BFAST-GATK and SHRiMP-GATK) can be delineated. In our study, a large fraction of variants identified only by a single approach had low quality (GATK LowQual filter value).

**Fig. 5:**
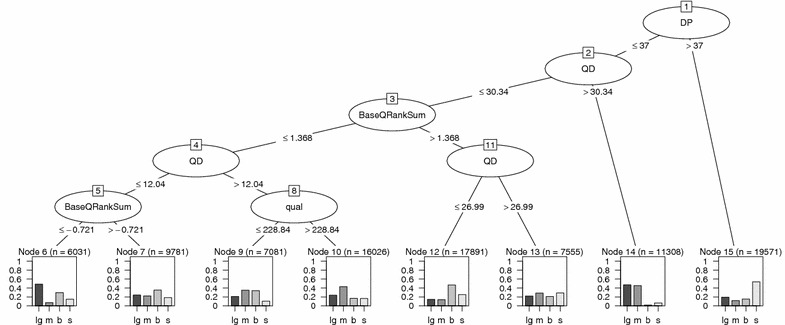
Decision tree diagram showing most discriminative annotations in classification of different categories of common variants identified in proband.* Rectangular terminal nodes* indicate fractions of variants (percentage) classified by the rules in each branch of the binary tree. Variable n indicates the number of samples from the training set assigned to that node. Node is associated with a class label of the most prevalent variant class. For example node 15 is associated with SHRiMP-GATK variants. Variant class identified by LifeScope-GATK is denoted by lg; SHRiMP-GATK is denoted by s; MAQ-GATK is denoted by m and BFAST-GATK by b. Tree nodes represented by ellipses show GATK variant annotations which were the most important in classifying the variants at each subsequent level. Classification rules are indicated by *less or equal than* and *greater than* conditions applied on a threshold value of the parameter. The diagram shows a considerable fraction of SHRiMP-GATK variants in node 15 characterized by larger depth of coverage (DP > 37). Large fraction of MAQ-GATK (m) variants are characterized by higher values of quality by depth (QD) (node 14) and have better GATK-assigned quality (see node 10). A group of LifeScope-GATK (lg) variants (node 14) are characterized by higher quality by depth (QD > 30.34). Another group (node 6) has lower QD and a negative value of BaseQRankSum, indicating poorer base quality support for alternative alleles. A tree size while running C50 algorithm was controlled constraining a split by minimum number of cases (parameter minCases) equal to 5000

**Table 7:** GATK steps

Step	Command	Description
Input BAM	*java -jar MarkDuplicates.jar INPUT = your_bam_file OUTPUT = step1.bam METRICS_FILE = Fmetrics_step1.bam ASSUME_SORTED = true*	Marking duplicates
Step 1.	*java -jar AddOrReplaceReadGroups.jar INPUT= step1.bam OUTPUT = step2.bam RGID= Read_Group ID RGLB = Read_Group_Library RGPL= platform RGPU = platform_unit RGSM= sample_name RGDS = Read_Group_Description RGDT = Read_Group_Run_Date*	Replacing all read groups in the INPUT file with a new read group
Step 2.	*java -jar ReorderSam.jar INPUT = step2.bam OUTPUT = step3.bam REFERENCE = ucsc.hg19.fasta*	Reorder reads in BAM file to match the contig ordering in a provided reference file
Step 3.	*java -jar SortSam.jar INPUT = step3.bam OUTPUT = step4.bam SORT_ORDER = coordinate*	Sorting the aligned reads by coordinate order
Step 4.	*java -jar BuildBamIndex.jar INPUT= step4.bam*	Generating BAM index
	*java -jar GenomeAnalysisTK.jar -T RealignerTargetCreator -R ucsc.hg19.fasta -S STRICT -I step4.bam -o indels.intervals -allowPotentiallyMisencodedQuals*	Indel Realignment I (Creating a target list of intervals to be realigned)
	*java -jar GenomeAnalysisTK.jar -T IndelRealigner -R ucsc.hg19.fasta -S STRICT -I step4.bam -targetIntervals indels.intervals -o step5.bam -known Mills_and_1000G_gold_standard.indels.hg19.vcf -known 1000G_phase1.indels.hg19.vcf -allowPotentiallyMisencodedQuals*	Indel Realignment II (Performing realignment of the target intervals)
Step 5.	*java -jar SortSam.jar INPUT = step5.bam OUTPUT = step6.bam SORT_ORDER = coordinate*	Sorting the aligned reads by coordinate order
Step 6.	*java -jar BuildBamIndex.jar INPUT = step6.bam*	Generating BAM index
	*java -jar GenomeAnalysisTK.jar -T BaseRecalibrator -I step6.bam -R ucsc.hg19.fasta -S STRICT -knownSites dbsnp_138.hg19.vcf -o recal.grp –covariate QualityScoreCovariate –covariate ReadGroupCovariate –covariate ContextCovariate –covariate CycleCovariate –solid_nocall_strategy PURGE_READ –solid_recal_mode SET_Q_ZERO_BASE_N -allowPotentiallyMisencodedQuals*	Base quality score recalibration I (data-driven adjustment of base quality scores)
	*java -jar GenomeAnalysisTK.jar -R ucsc.hg19.fasta -S STRICT -I step6.bam -T PrintReads -o step7.bam -BQSR recal.grp -allowPotentiallyMisencodedQuals*	Base quality score recalibration II (Applying the recalibration to sequence data)
Step 7.	*java -jar GenomeAnalysisTK.jar -R ucsc.hg19.fasta -T HaplotypeCaller -I step7.bam -S STRICT –dbsnp dbsnp_138.hg19.vcf -minPruning 3 -o step8.vcf -stand_call_conf 50 -stand_emit_conf 30*	Calling variants in sequence data
Step 8.	*java -jar GenomeAnalysisTK.jar -R ucsc.hg19.fasta -T SelectVariants –variant step8.vcf -o step9_SNP.vcf -selectType SNP -S STRICT*	Select SNPs from the input file
Step 9.	*java -jar GenomeAnalysisTK.jar -T VariantRecalibrator –input step9_SNP.vcf -R ucsc.hg19.fasta -S STRICT -resource:1000G,known = false,training = true,truth = false,prior = 10 1000G_phase1.snps.high_confidence.hg19.vcf -resource:hapmap, known =f alse, training = true, truth = true, prior = 15.0 hapmap_3.3.hg19.vcf -resource:omni, known=false, training = true, truth = true, prior = 12.0 1000G_omni2.5.hg19.vcf -resource:dbsnp, known = true, training = false, truth = false, prior = 2.0 dbsnp_138.hg19.vcf -an QD -an MQRankSum -an ReadPosRankSum -an FS -an MQ –maxGaussians 4 -mode SNP -recalFile recal -tranchesFile tranches*	Building SNP recalibration model
	*java -jar GenomeAnalysisTK.jar -R ucsc.hg19.fasta -T ApplyRecalibration -S STRICT –input step9_SNP.vcf -ts_filter_level 99.5 -mode SNP -tranchesFile tranches -recalFile recal -o step10_final.vcf*	Applying SNP recalibration model

Which of variant annotations discriminate the variant classes the best, was explored using common variants by C5.0 decision tree algorithm [28] in R. We do not attempt to fit a classification model but rather to perform exploratory analysis to discover thresholds of variant annotations best discriminating variant classes. C5.0 algorithm learns this information from data. A most discriminative annotation was root mean square mapping quality value (MQ). Almost all SHRiMP-GATK variants were assigned to a class characterized by high MQ ($>99$). Mapping quality computation is specific to each mapping program. Therefore, MQ values might not be directly comparable between the approaches. MQ and MQRankSum were exluded from the exploratory list of annotations and the additional sets of rules discriminating the variants were identified. Class of SHRiMP-GATK variants had a larger value of depth of coverage (DP). Class of MAQ-GATK variants was characterized by higher value of quality by depth (QD) and better quality assigned by GATK. One subclass of Lifescope-GATK variants had higher QD values. Another subclass of Lifescope-GATK variants had lower QD and a negative BaseQRankSum, indicating poorer base quality support for alternative alleles. The diagram of a decision tree of these classifications is shown in Fig. 5.

Using LifeScope BAM files, a variant calling was performed by both LifeScope and GATK. Some variants were identified solely by LifeScope and vice versa. In the GATK variant calling pipeline an adjustment of reported base qualities in BAM file is performed according to the estimated empirical base quality scores (see step 8 in Table 7). We observed that some base qualities reported in LifeScope’s BAM file were diminished and some were elevated by GATK’s base recalibration procedure. This adjustment resulted in some sites called variants by GATK but not by LifeScope and vice versa. The sites called variants by LifeScope were not recognized by GATK because of reduction of base quality scores resulting from the base recalibration. Default variant calling parameters of LifeScope are less stringent (for example a minimum coverage for Heterozygote call is 2). Therefore such variants identified by LifeScope are filtered by GATK.

### Shortcomings of used alignment programs

Our aim was to assess performances of the tools with the default parameters. A major shortcoming of MAQ was rather long time required to complete analysis since MAQ was not designed to utilize multicore computing resources. MAQ and BFAST generally produced lower coverage of targeted regions. However, their alignments had higher mapping quality overall. SHRiMP was the best in terms of speed and coverage. However, with the default parameters SHRiMP produced alignments characterized by increased number of polymorphic sites. This influenced an increased number of false calls accounted for by a presence of at least one alternative allele observed in BAM files of other mapping programs. LifeScope, albeit fully utilizing available multicore architecture, also had long running time. Another shortcoming of LifeScope is a lack of standalone tools similar to FastQC allowing to perform initial quality analysis of sequenced reads compressed in XSQ files.

## Conclusions

We provided a comprehensive study of family exomes obtained by AB SOLiD 5500 platform and contributed technical details of the used pipelines. Our performed analysis strongly suggests that LifeScope’s proprietary mapping program is the best choice for processing color-space coded data generated by AB SOLiD platform. Although our conclusion was anticipated we nevertheless provided quantitative analysis to support it. In all cases LifeScope’s pipeline stood out in terms of achieving high coverage and providing high confidence variants.

Coverage is one of the most important factors in calling a variant. Variant calls with low coverage might not indicate a true variant in the exome. Nevertheless a high coverage not necessarily indicates a true variant as well. A caveat was encountered in interpreting high coverage variants detected by SHRiMP-GATK approach. Larger than expected amount of high coverage singleton variants identified by SHRiMP-GATK that did not appear in the dbSNP database of known variants was observed. This prompted to a variant assessment strategy by exploring mappings (pileups [27]) in alternative BAM files at the genomic positions in which an unknown singleton SHRiMP-GATK variant was detected. We tested if a singleton variant is supported by the evidence of a presence of at least one alternative allele in that position in at least two other mappings. If it was not, the variant was filtered out.

This study was intended to explore near-default settings of the mapping programs, therefore a parameter optimization was not attempted. Aforementioned variant assessment strategy was applied only to SHRiMP-GATK variants since other pipelines did not deviate considerably from the expected small counts of singleton variants that are not present in dbSNP. In this study we used adjusted set of SHRiMP-GATK variants.

Our study reveals practical benefits of aggregating variant calling results of several pipelines. First of all variants that were identified by several methods have higher confidence of being truly present in the exome. If in doubt whether to consider the variant for further analysis because of its poorer mapping quality and coverage, one can gain confidence by examining mapping profile of the same genomic position in the alignment generated by some alternative mapping program. In summary:

- LifeScope’s proprietary pipeline is method of choice for analysis of color-space coded sequencing data generated by AB SOLiD 5500 platform. LifeScope provided superior coverage of the exome sequencing data.
- Confirmation. Use of alternative pipelines may help in assessing an insufficiently covered variant and increasing the confidence about this identified variant truly being present in the exome.
- Sensitivity. Uniting lists of variants identified by several alternative mapping and variant calling pipelines allows to identify important deleterious variants that might have been missed by a single method because of poor coverage or mapping quality at that position.
- Choice of coverage threshold should be data-driven. Coverage of variants is not uniform. Due to this reason setting the high threshold on coverage may induce a considerable loss of important variants that may be strongly related to the manifestation of a phenotype of interest.

## Methods

A workflow of exome analysis by LifeScope and the alternative pipeline is presented in Fig. 1. The raw exome data obtained from the sequencer in XSQ format is aligned to the reference genome by LifeScope mapping program. Alternatively, the XSQ files are converted to color-space fastq, csfasta and quality files and subsequently are aligned to the reference genome by MAQ, SHRiMP and BFAST mapping programs. In this way one exome is represented by four BAM files generated by four alignment tools. Variant calling is performed by two methods: LifeScope’s diBayes algorithm and Genome Analysis Toolkit (GATK).

### LifeScope computational pipeline

**Table 8:** Parameters in LifeScope’s secondary and tertiary analysis software modules

	Default value
Alignment parameters	
Minimum number of non-matches allowed during indel finding	9
Maximum deletion size (in a gapped alignment in the first pass)	19
Maximum insertion size (in a gapped alignment in the first pass)	4
The minimum edge length required for insertions and deletions on the first pass	12
Number of mismatches allowed for gap alignments	3
Minimum mapping quality value (MAPQ) allowed for aligned read	8
Minimum edge length required for insertions and deletions	12
The seed window side allowed to the left of the anchor alignment	40
The seed window side allowed to the right of the anchor alignment	80
Maximum number of alignments for a read on the first pass which gives the maximum number of hits that are reported in the mapping output	50
SNPs analysis module using diBayes algorithm variant calling parameters	
Minimum allele ratio (Het)	0.15
Minimum coverage (Het)	2
Minimum non-reference base QV (Het)	28
Minimum average non-reference base QV (Hom)	28
Minimum base quality value for a position	28
Minimum base quality value of the non-reference allele of a position	28
Mapping quality value of the read	$>$8
SNP call stringency. Alleles on both strands	Not required
Threshold of mismatch/alignment-lengh ratio	1
Base candidate allele quality value	$>$7
Minimum number of unique start positions required to call heterozygote, homozygote	2
Proportion of the total reads containing either of the two candidate alleles	0.65

Exome analysis by Life Technologies™ LifeScope™ 2.5.1 genomic analysis software was carried out in three stages described as primary, secondary and tertiary analyses [13]. Primary analysis of image acquisition and bead processing, application of quality metrics and color calls was performed within the SOLiD sequencer. Secondary and tertiary analyses were performed by LifeScope software using targeted.resequencing.frag workflow. This workflow consists of 7 LifeScope software modules. SAET, Mapping and Mapping statistics (BAMStats) are secondary analysis modules. Modules of tertiary analysis are Enrichment, SNPs, Small indels and Annotations. Default parameter values were used in LifeScope analysis as shown in Table 8.

### Alternative pipeline to the LifeScope

**Table 9:** List of open source tools used in our study of the alternatives to the LifeScope pipeline

Analysis category	Tool	Web reference
Convert XSQ to color-space fastq	XSQtools solid2fastq	Life Technologies website Bfast package
Alignment to the reference genome	Maq v.0.7.1	http://maq.sourceforge.net/
	Default parameters, -n 3	
	Shrimp 2.2.3	http://compbio.cs.toronto.edu/shrimp/
	Default parameters, -h 85 –strata -o 3	
	Bfast 0.7.0	http://sourceforge.net/projects/bfast/
	Default parameters	
Preprocessing	Picard 1.111	http://picard.sourceforge.net/
	Samtools 0.1.18	http://samtools.sourceforge.net/
Variant calling	GATK v.3.1	http://www.broadinstitute.org/gatk/
	Steps and parameters in Table 7	
Variant summaries	Vcftools 0.1.12	http://vcftools.sourceforge.net/
	Bcftools v.0.2.0	http://samtools.github.io/bcftools/
Variant annotation	Annovar	http://www.openbioinformatics.org/annovar/

Components of a pipeline alternative to the LifeScope are summarized in Table 9. The sequenced exomes in the SOLiD’s XSQ file format were transformed to the color-space fastq format by XSQTools and BFAST solid2fastq converters. Transformed reads were mapped to human hg19 reference genome by three alignment programs MAQ [29], SHRiMP [9] and BFAST [30]. Inputs for MAQ were color-space csfasta and quality qual files. SHRiMP and BFAST alignment programs used color-space fastq files as inputs. SHRiMP and BFAST were chosen because of their ability to utilize a multiple-core architecture of available computational resources. The MAQ aligner was included because of its stringency and mapping accuracy reported in benchmarks [31]. By allowing three mismatches in MAQ a number of mapped reads increased by up to 10 % at the expense of a threefold mapping time increase. In SHRiMP the default value of -h parameter controlling the quality of mapping window was changed to 85 %. In BFAST default parameter values were used. Variant calling was performed by Genome Analysis Toolkit (GATK) pipeline using BAM files generated by LifeScope, MAQ, SHRiMP and BFAST aligners as input. Variant calling workflow by GATK is presented in Table 7.

### Algorithmic details of the mapping programs

Programs mapping next generation sequencing reads to the reference genome are subdivided into several categories depending on how indexing and string matching are organized. They consist of algorithms that use a hash table (MAQ, BFAST, SHRiMP) and algorithms that use suffix trees (Burrows Wheeler Algorithm) [32]. Mapping programs used in the current study use hash tables for indexing of color-space encoded reference. MAQ encodes the reference genome into internal format. In the alignment phase MAQ indexes the reads. BFAST, SHRiMP and LifeScope index the reference genome using spaced seeds. All mappers perform gapped alignment, essential for a discovery of single nucleotide variations. In alignment of reads BFAST and SHRiMP at first finds a global list of candidate alignment locations for each read. During a second pass, the Smith-Waterman algorithm is applied locally on the identified candidate locations to find the best hits matching the reads [8]. Scholarly treatment of dynamic programming algorithm for sequence alignment that underlies local sequence alignment in used mapping programs can be found in the reference [33] on pages 172–176. In LifeScope mapping program a global indel finding extension option is used for alignment, which extends from anchor alignment and does a full length gapped extension with the allowed number of mismatches (set to 3) [13].

## Abbreviations

BFAST: Blat-like Fast Accurate Search Tool
COSMIC: Catalog of Somatic Mutations in Cancer
DP: depth of coverage
GATK: Genome Analysis Tool Kit
MAQ: Mapping and Assembly with Qualities
NGS: Next Generation Sequencing
QUAL: Quality
SHRiMP: Short Read Mapping Package
SNP: Single Nucleotide Polymorphysms
Ti: transition
Tv: transversion
